# P-1782. Antimicrobial Stewardship Program Resourcing across Hospitals over Time: A Repeated Cross-Sectional Study

**DOI:** 10.1093/ofid/ofae631.1945

**Published:** 2025-01-29

**Authors:** Valerie Leung, Sera Thomas, Kevin Brown, Nick Daneman, Kevin Schwartz, Bradley J Langford

**Affiliations:** Public Health Ontario, Toronto, Ontario, Canada; Public Health Ontario, Toronto, Ontario, Canada; University of Toronto, Toronto, Ontario, Canada; Public Health Ontario, Toronto, Ontario, Canada; Public Health Ontario Dalla Lana School of Public Health, University of Toronto, Toronto, Ontario, Canada; Public Health Ontario, Toronto, Ontario, Canada

## Abstract

**Background:**

Antimicrobial stewardship programs (ASP) are a crucial component of an overarching One Health approach to mitigate antimicrobial resistance (AMR). Adequate resourcing is a predictor of success for hospital ASPs. To understand the progress of hospital ASPs, we conducted periodic surveys tracking the level of resourcing over time.

**Figure:**
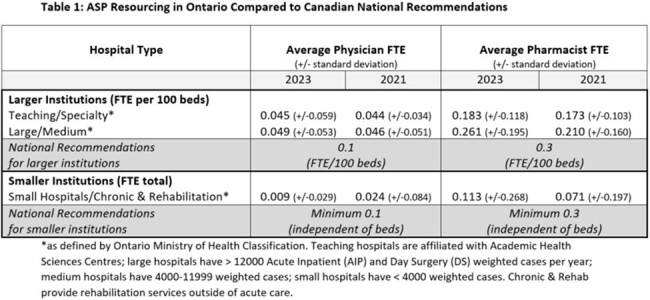

**Methods:**

Public Health Ontario’s Hospital ASP Landscape survey is conducted every 2-3 years, beginning in 2016 and most recently in 2023. This online survey is disseminated using a targeted distribution list to reach antimicrobial stewardship practitioners in acute care and complex continuing care & rehabilitation hospitals. Descriptive analysis was performed at an aggregate level and by hospital type. Full-time equivalent (FTE) staffing ratios were compared to Association of Medical Microbiology and Infectious Disease (AMMI) Canada recommendations for 2021 & 2023.

**Results:**

In 2023, the survey response rate was 70% (90/129) of hospitals with 98% reporting the presence of a formal ASP. Response rate of previous surveys ranged from 55-78%. In 2023, the proportion of organizations reporting any designated resources to support their program was 60%; this was 50% in 2016, 57% in 2018 and 53% in 2021. The proportion of hospitals meeting the AMMI resourcing recommendations for physician and pharmacist FTE in 2023 was 10% and 21% compared with 7% and 13% in 2021. For teaching hospitals, the average physician FTE/100 beds was 0.045 in 2023 and 0.044 in 2021; the average pharmacist FTE/100 beds was 0.183 in 2023 and 0.173 in 2021. For large & medium hospitals, average physician FTE/100 beds was 0.049 in 2023 and 0.046 in 2021; the average pharmacist FTE/100 beds was 0.261 in 2023 and 0.210 in 2021. For small & rehabilitation hospitals, the average physician FTE was 0.009 in 2023 and 0.024 in 2021; the average pharmacist FTE was 0.113 in 2023 and 0.071 in 2021. (Table 1)

**Conclusion:**

The proportion of hospitals reporting any designated resources to support their ASP is relatively unchanged since 2016. Resource allocation continues to be below national recommendations for physician and pharmacist FTEs, especially for smaller institutions.

**Disclosures:**

**All Authors**: No reported disclosures

